# Transgenerational Effects of Traumatic Historical Events on the Incidence of Metabolic Syndrome/ Nonalcoholic Fatty Liver Disease in the Romanian Population

**DOI:** 10.25122/jml-2020-0156

**Published:** 2020

**Authors:** Victor Stoica, Daniel Adrian Gardan, Ileana Constantinescu, Iuliana Petronela Gardan, Bogdan Calenic, Mircea Diculescu

**Affiliations:** 1. Department of Gastroenterology, “Carol Davila” University of Medicine and Pharmacy”, Bucharest, Romania; 2. Department of Gastroenterology, Fundeni Clinical Institute, Bucharest, Romania; 3. Faculty of Economic Sciences, Spiru Haret University, Bucharest, Romania; 4. Department of Immunology and Transplant Immunology, “Carol Davila” University of Medicine and Pharmacy, Bucharest, Romania; 5. Center of Imunogenetics and Virusology, Fundeni Clinical Institute, Bucharest, Romania; 6. Department of Biochemistry, Faculty of Dentistry, “Carol Davila” University of Medicine and Pharmacy, Bucharest, Romania

**Keywords:** Metabolic disease, lifestyle, traumatic event, transgenerational epigenetic inheritance

## Abstract

Concerns for successful public health management are integrated into the core business of government-responsible institutions. Diseases associated with metabolic syndrome are very common in the Romanian population. In our study, we focused on the cardiovascular and non-alcoholic fatty liver disease (NAFLD). The article starts from the hypothesis that the increased incidence of such diseases is determined today by the cumulative effect of traumatic historical events such as the famine of 1946-47 and the communist political regime specific to the 80s and 90s. This study aims to present the arguments that indicate the correlation of economic variables whose variation can be easily determined by traumatic events that affected the economy, with variables able to measure the incidence of various diseases usually associated with metabolic syndrome or NAFLD. A series of statistical data were analyzed from the official sources available in the form of consecutive value data for the 1995-2018 period. The results highlighted a direct and strong link between the variable gross domestic product (GDP) per capita in USD, 2011 purchasing power parity (PPP) and specific incidence of circulatory, nutritional endocrine and metabolic diseases, as well as a strong and inverse link between GDP and infant’s deaths per 1000 live births. Conclusions highlight that the effects of traumatic historical events must be made aware through medical education of the population, supporting the idea according to which the incidence of various metabolic diseases is greater for the offspring of those who have actively suffered during such events.

## Introduction

Romania is one of the countries which, just thirteen years ago, became a member of the European Union (EU), and since then, it is struggling to evolve respecting communitarian rules.

Even before the admission, the country faced an exodus of the active population towards Occidental countries searching for a higher income and, why not, better healthcare and educational system. We expect a continuous decrease in the active Romanian population in the context of migration, decreased birthrate, a dramatic change in the population structure with a tendency towards aging. We also expect increased costs for chronic illnesses.

The overall equation is very complicated with increasing costs for healthcare from one year to another at the same time with anticipated decreasing contributions from the diminishing of the active segment of the population.

During the same study period, the prevalence of obesity among Romanians increased from 19.1% in 2007 [1] to 31.6% in 2016 [2]. Obesity is highly correlated with diseases such as diabetes, non-alcoholic fatty liver disease (NAFLD) and cardiovascular diseases [3]. Therefore, any increase in obesity prevalence generated by an unhealthy lifestyle will be associated with corresponding increases in the prevalence of NAFLD and other chronic conditions. Although, generally speaking, the increased number of diagnostic methods may increase the number of treated patients [4], nowadays, we encounter a high prevalence of chronic diseases. Nevertheless, does the increase in income and unhealthy lifestyle explain the epidemic of chronic diseases in Romania during the last 30 years? Alternatively, is there another mechanism related to our recent and past history of highly traumatic events?

Regarding the matter of NAFLD, some important details have to be mentioned. Recently, an international panel of experts recommended using the term metabolic dysfunction-associated fatty liver disease (MAFLD) instead of NAFLD as a way of representing the hepatic manifestation of a multisystem disorder. The new definition for NAFLD includes criteria such as obesity (associated in most cases with a fatty liver), type 2 diabetes mellitus (as >70% of patients with type 2 diabetes mellitus have MAFLD) but also includes lean/normal-weight patients with metabolic disorders (prediabetes, dyslipidemia, high blood pressure, pro-inflammatory status revealed by an elevated C-reactive protein) [5].

The high prevalence of MAFLD can be explained by several pathogenic factors: the imbalance between ingested calories and physical activity, genetic polymorphisms (especially patatin-like phospholipase domain-containing protein 3 - PNPLA3 and transmembrane 6 superfamily member 2 - TM6SF2), overproduction of reactive oxygen species, ectopic lipid accumulation in non-adipocyte cells, intestinal dysbiosis. Except for diet and vigorous physical exercise, there is no other effective therapy for MAFLD. However, we have to treat dyslipidemia or diabetes in order to prevent the appearance of significant cardiovascular or metabolic complications, with no significant effect on liver fat accumulation and inflammation [6].

Our study correlates for the first time Romania’s economic indicators and quantifies the Effect of Traumatic Events on Population (ETEP) with medical indicators such as infant mortality rate and prevalence of metabolic (MAFLD, diabetes) and cardiovascular diseases. This study proposes a statistical model able to explain the actual burden of chronic diseases in Romania through possible epigenetic changes induced by prolonged traumatic events (the famine of ’46-’47 and the “scientific program for healthy living” - organized famine in the ‘80s).

Our conclusion is that the Romanian population can be considered at high risk for several chronic illnesses not only due to an unhealthy lifestyle but also because of historical events with transgenerational effects.

## Material and Methods

In order to validate the hypothesis according to which the increased incidence of such diseases is currently determined by the cumulative effect of some traumatic historical events, the analysis of consecutive time series from official sources was taken into account. Thus, the data regarding the incidence of the analyzed diseases were taken from statistical yearbooks of the National Institute of Public Health, National Center for Statistics and Informatics in Public Health [7, 8]. Regarding the data related to the gross domestic product (GDP) per capita in USD, 2011 PPP values at comparable prices, the information provided in the volume “A century of sincerity. Recovering the lost memory of the Romanian economy 1918-2018” edited by Liviu Voinea was taken into account [9]. Calorie consumption data were taken from the statistical yearbooks of Romania published by the National Institute of Statistics [10, 11] and Eurostat statistics [12]. Thus, to identify the existence of a correlation between GDP/capita in USD, 2011 purchasing power parity (PPP) and infant’s deaths per 1000 live births, GDP and specific incidence of circulatory diseases, GDP and specific incidence of nutritional endocrine and metabolic diseases, official databases were used for a period of 24 years (1995-2018). It should be noted that the authors were limited by the lack of consecutive data series for a longer period of time when conducting the study.

The analysis of the statistical data was performed using the SPSS 26.0 software, calculating the parameters of the regression equations and the necessary statistical tests in order to validate the identified regression models.

## Results

As we have already mentioned, our intention was to correlate economic indicators with healthcare ones. It is well known that the infant mortality rate is lower in countries developed economically. Romania had one of Europe’s highest infant mortality rates in 1970 (49.49‰). During the fall of communism, in 1989, the infant mortality rate was 26.9‰ [7].

The crash of a centralized economy led to a dramatic drop in GDP, depression, hyperinflation, and decreased purchasing power during the ’90s. Just in the early 2000s, the GDP rose to the level from 1989-1990.

In order to test our working hypothesis regarding the effect of traumatic historical events on MAFLD-related conditions, we took into consideration the GDP as a reliable measurement for the economic inputs and three different variables as measurements regarding disease prevalence. All the data have been computed for the period 1995-2018 because it was the only period available with reliable statistical records for all the variables implied and from the point of view of adequate measurement (official statistics inputs, data for consecutive years).

We began to run the data analysis of the available statistic series using the SPSS software (version 26.0). We considered the relationship between GDP as an independent variable and infant mortality rate per 1000 live births, the specific incidence of circulatory diseases, specific incidence of nutritional endocrine and metabolic diseases as dependent variables, and we implemented a regression equation for every relationship. The results computed for the regression equation (Y = B_0_ + B_1_X_1_ + Ƹ) are presented in Tables 1, 2 and 3.

**Table 1:** Model summary for correlation between GDP and infant’s mortality rate, the specific incidence of circulatory diseases and the specific incidence of nutritional endocrine and metabolic diseases (the analyzed period was 1995-2018).

Model	R	R Square	Adjusted R Square	Std. Error of the Estimate	Change Statistics
						R Square Change	F Change	df1	df2	Sig. F Change
1	.965^a^	.931	.928	1.4639	.931	297.342	1	22	.000
2	.852^a^	.726	.713	497.1876	.726	58.201	1	22	.000
3	.935^a^	.874	.868	333.8363	.874	152.376	1	22	.000

1. a. Predictors: (Constant), GDP/capita; b. Dependent Variable: Infant’s deaths per 1000 live births; 2. a. Predictors: (Constant), GDP/capita; b. Dependent Variable: Specific incidence of circulatory diseases – new cases per 100,000 inhabitants; 3. a. Predictors: (Constant), GDP/capita; b. Dependent Variable: Specific incidence of nutritional endocrine and metabolic diseases - new cases per 100,000 inhabitants. Data source [7-9].

**Table 2:** ANOVA statistics regarding the variables considered.

Model		Sum of Squares	df	Mean Square	F	Sig.
1	Regression	637.204	1	637.204 2.143	297.342	.000^b^
	Residual	47.146	22			
	Total	684.350	23			
2	Regression	14387051.997	1	14387051.997 247195.494	58.201	.000^b^
	Residual	5438300.863	22			
	Total	19825352.860	23			
3	Regression	16981817.308	1	16981817.308 111446.680	152.376	.000^b^
	Residual	2451826.965	22			
	Total	19433644.273	23			

**Table 3:** Coefficients’ statistics regarding the variables considered.

Model		Unstandardized Coefficients		Standardized Coefficients	t	Sig.	95.0% Confidence Interval for B	
		B	Std. Error	Beta			Lower Bound	Upper Bound
1	(Constant)	31.940	1.106	-.965	28.868	.000	29.646	34.235
	GDP	-.001	.000		-17.244	.000	-.001	-.001
2	(Constant)	786.540	378.182	.852	2.080	.049	2.239	1570.841
	GDP	.184	.024		7.629	.000	.134	.234
3	(Constant)	-1198.885	253.930	.935	-4.721	.000	-1725.504	-672.267
	GDP	.200	.016		12.344	.000	.167	.234

1. Predictors: (Constant), GDP/capita; Dependent Variable: Infant’s deaths per 1000 live births; 2. Predictors: (Constant), GDP/capita; Dependent Variable: Specific incidence of circulatory diseases - new cases per 100,000 inhabitants; 3. Predictors: (Constant), GDP/capita; Dependent Variable: Specific incidence of nutritional endocrine and metabolic diseases - new cases per 100,000 inhabitants.

From the point of view of the first relationship, between GDP and infant’s deaths per 1000 live births, it was not such a great surprise to obtain an R2 value of 0.931, indicating a very strong, negative correlation between GDP/capita and infant mortality rate. Despite the economic downturn in the ’90s, the trend of decreasing infant mortality rate continued. For us, this indicator is very important as it provides at a glance a huge amount of information regarding the ability of governments to implement efficient healthcare policies, to use the financial resources efficiently, the decrease of the proportion of “unwanted” babies, population access to preventive and curative healthcare services. Pearson’s correlation coefficient (Pearson Correlation = -.965, Sig. = .000) indicates a strong inverse correlation between the analyzed variables; as GDP decreases, the number of deaths under 1 year increases. The coefficient of multiple determination (R square) measures the intensity of the relationship expressed by the regression equation. In other words, 93.1% of the variation of the dependent variable (infants deceased under 1 year) is determined by the variation of the cause variable (GDP), and only 6.9% due to the variation of the residual variable.

It is also important to note the difference between R square and adjusted R square because it shows the degree of generalization of the regression model in the entire research population. In this case, if the regression model were to be extended to the entire research population, the variation of the dependent variable would decrease by .003 (less than 1 percentage point) compared to its variation encountered in the sample studied. The F Change statistical test has a good value, so the value obtained is statistically significant. There is a very small probability that the value obtained is due to chance and not to a real connection (because Sig. F Change = .000, which is a value below .01).

After demonstrating the correlation between infant mortality rate and GDP/capita, we thought about searching the correlation between GDP/capita and the incidence of cardiovascular diseases. Several clinical trials have shown a dramatic increase in the incidence of cardiovascular diseases in Romania but with a trend of stabilization or even declining in the last few years. We have tested the possible correlation between GDP and incidence of cardiovascular disease and the results are showing a strong, direct relationship between the GDP/capita and the incidence of cardiovascular diseases. The R2 value of 0.726 provides us the valuable information that 72.6% of the variation in the incidence of cardiovascular diseases can be explained by the GDP/capita variation. Anecdotal reports, personal clinical experience, and clinical trials made by Romanian colleagues specialized in nutrition diseases claim a dramatic increase in the incidence of obesity, diabetes, and MAFLD.

Pearson’s correlation coefficient (Pearson correlation = .852, Sig. = .000) also indicates for this relationship a strong correlation between the analyzed variables; as the GDP increases, the incidence of cardiovascular diseases increases. For this correlation, 72.6% of the variation of the dependent variable (specific incidence of circulatory diseases) is determined by the variation of the cause variable (GDP), and only 27.4% due to the variation of the residual variable.

For the last correlation, we tested if the increase in GDP/capita can be correlated with the incidence of nutrition diseases. We can assess a strong enough correlation between the two variables; a demonstration of the fact that as income increases in a developing economy, people will have the tendency of consuming more than needed and, in a matter of years, the consequences of such behavior are reflected by the steep increase in the incidence of nutritional diseases. The R2 value of 0.874 and Pearson’s correlation coefficient (Pearson correlation = .935, Sig. = .000) confirm a strong positive correlation between the two variables considered.

**Figure 1:**
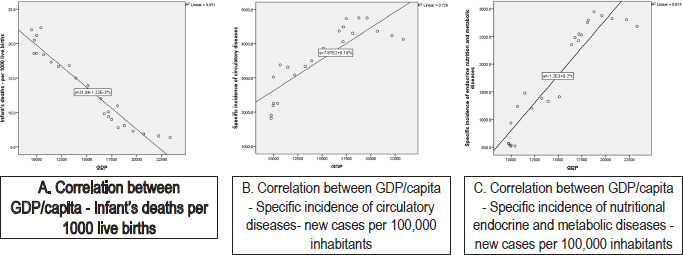
Regression equations corresponding to the correlations considered.

Figure 1 shows the regression equations for the three correlations considered, the sign of the estimated parameters showing the way in which the independent variable influences the dependent variable. The negative sign in the case of the first correlation indicates the influence of the GDP variable on deceased children under 1 year of age, while in the case of the other two correlations, the sign of the estimated parameters being positive, shows a direct relationship between GDP and the specific incidence of circulatory diseases, and the specific incidence of nutritional endocrine and metabolic diseases.

The population group of age between 65-74 years represents the ones born between 1940 and 1949. Data from the 2019 European Obesity Day (EOD) report shows that for this particular group of age, the proportion of obese people is 71.2% in 2014, being the highest value in comparison with any other age group in Romania [13]. For the group between 45 to 64 is 69.3%, and for the group of 35-44 is 54.4%. It can be observed that the generations that have been born during the great Romanian famine from 1946-1947 that was developing especially in the Moldavia region and the actual territory of the Republic of Moldova belong to this age group. At the same time, another correlation can be made regarding the age group of 18 years and above that are the grandchildren of the ones born in 1946-1947. One of the clinical trials published by our colleagues Mota et al. - the PREDATORR study - reveals “the unexpectedly high prevalence of obesity/overweight, abdominal obesity, and metabolic syndrome (MetS) in the adult Romanian population”. [2] We must mention the relatively high prevalence of obesity (20.9%) and MetS (20%) in the 20-39 age group [2].

Another interesting line of analysis that can be performed refers to the fact that the appropriate expression of the transgenerational effects that traumatic historical events can have on the incidence of diseases associated with metabolic syndrome/MAFLD can be optimally achieved with the help of several aggregate indicators that, unfortunately, are not yet calculated or not covered for longer periods (50-60 consecutive years) in our country.

**Figure 2:**
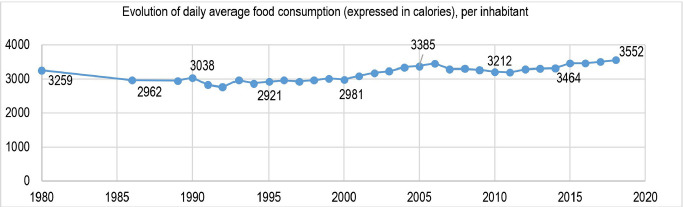
Evolution of daily average food consumption (expressed in calories), per inhabitant. Data source: [11].

One statistic that may be relevant in this context is that of calorie intake. From the available data [10, 11], a constant increase in the volume of calories consumed, especially after 2001, is visible. It is a phenomenon that manifested against the background of market liberalization and change in consumer behavior population in the direction of consuming a diversity of products, a natural reaction to the deprivations existing before 1989 (Table 4, Figure 2).

**Table 4:** Evolution of daily average food consumption (expressed in calories), per inhabitant.

1980	1986	1989	1990	1991	1992	1993	1994	1995	1996	1997	1998	1999	2000	2001	2002
3259	2962	2949	3038	2832	2758	2959	2872	2921	2953	2933	2959	3020	2981	3092	3179
2003	2004	2005	2006	2007	2008	2009	2010	2011	2012	2013	2014	2015	2016	2017	2018
3233	3350	3385	3455	3290	3300	3273	3212	3199	3287	3302	3320	3464	3462	3500	3552

During the last 30 years, the total calorie intake is higher than the recommendations of the Food and Agriculture Organization (FAO) of 2700 calories/day in a temperate climate condition. The amount of food purchased by Romanians is excessive, with significant food waste. This behavior can also be explained by a transgenerational effect of food deprivation during previous times [14].

The situation is even more dramatic if we take into account the daily intake of calories per capita depending on the source [12]; in the case of the Romanian population, there are even higher calorie values, which indicates that Romanians buy food in an even higher percentage than the one corresponding to the amount of food consumed. Again, this behavior indicates the possibility of a transgenerational effect of food deprivation from previous periods.

The food consumption behavior of Romanians has a series of peculiarities that, in addition to possible transgenerational influences, favor the appearance and manifestation of a whole range of diseases, of which metabolic and cardiovascular diseases are the most prevalent. Thus, we are talking about a high share of food expenditures in total consumption expenditures (about 55.8% in 2001 - a year with significant economic difficulties compared to a percentage between 40-50% in the years considered more prosperous) [14]; excessive consumption of foods lacking high nutritional qualities such as cereals and potatoes which predispose to diabetes under stress; consumption well above the allowable limit of alcohol, tobacco and fats which are conducive to cardiovascular diseases, cancer, tuberculosis, and MAFLD; a proportion of about 2.5% of the total population that is affected by chronic malnutrition; a daily food basket consisting of small proportions based on local ecological products for the vast majority of the population in the urban area, which contrasts with the high share of agricultural products from own production consumed by the population in the rural area [14].

Our data suggest the influence of psychological distress on the incidence of MetS and MAFLD. In this regard, improvements are needed on Inquiries on Life Quality and on Statistics on Income and Living Conditions, all processes triggered by Romania’s admission to the EU.

## Discussion

### Examples of traumatic historical events – famine/deportation

Traumatic historical events are considered events that can produce effects on a certain population resulting in long-standing and intergenerational adverse outcomes. [15]

Some of the well-known and documented traumatic historical events are represented by the Holocaust, the “Dutch Hunger Winter”, the siege of Leningrad and others. Offsprings of the Holocaust survivors are at high risk of anxiety, depression, posttraumatic stress disorder (PTSD), and cardiovascular events compared to the unexposed Jewish population [16, 17].

The Dutch Hunger Winter was a five-month period of acute food shortage from November 1944 until April 1945. As a retaliation for the railroad workers’ strike, the Nazi occupants blocked the food supplies to the population, a phenomenon aggravated by the canals’ freezing. During that period, children were born to malnourished mothers. The consequence of such an event after 20-30 years was a substantial increase in the cardiovascular risk (some say that it was three times greater), higher incidence of glucose intolerance, and atherogenic profile among exposed individuals [18].

The Siege of Leningrad was another highly traumatic event for the Russian population of Leningrad, which was isolated and malnourished due to Nazi occupation for two and a half years (September 1941 to January 1943). The less documented event seemed to lead to a higher risk of hypertension and cardiovascular disease among the offsprings of survivors.

Another highly traumatic but undebated such event was the brutal communist regime in Romania, one of the toughest in Eastern Europe. After the end of World War II, Romania was under Soviet occupation. Most of the country’s resources were drained by the Soviets as war compensations. Superimposed on that, during the’ 46-’47s came a severe drought that led to severe famine and even to death by hunger in some Romanians regions. All the children that were born in such difficult times had health-related issues later in adulthood. The period that followed highlights an evolution far from normal standards.

According to previous experiences mentioned above regarding other famines events, we should have expected a rise in the incidence of obesity, fatty-liver disease, cardiovascular disease during the’ 70-’80s in the affected Romanian population. However, what happened in Romania in that period was, unfortunately, an unprecedented social experiment. The dictatorship regime led by Nicolae Ceausescu failed to ensure the population the most basic goods, including food. The official propaganda stated that Romanians were better fed than Americans (3300 calories/individual) [19]. At that time, Romania exported most of its food production in order to pay the external debt, and the performance of Romanian agriculture was very poor. The real situation was well-known to Nicolae Ceausescu; a report of State Security stated that, in December 1989, the situation of the food supply in Bucharest was the following [19]:

• Meat - 345 tones needed, only 212 tones delivered (27.8%);
• Dairy products - 109 tones needed; only 30 tones delivered (27.5%);
• Vegetables - 871 tones needed; only 246 tones delivered (28.2%);
• Potatoes - 1258 tones needed, only 358 tones delivered (28.4%);
• Rice - 129 tones needed; only 11 tones delivered (8.5%).

Bucharest was the capital of Romania, and it should have been better supplied than other cities across the country; therefore, we can imagine the situation of smaller Romanian cities. Food rationing was introduced officially from 1982 until the fall of the communist regime in 1989 as a “scientific program for healthy eating” [19].

At that time, the infant mortality rate in Romania was one of the highest in Europe. In our study, we considered the infant mortality rate as one of the most important and complex indicators. It reflects the mother’s health and nutrition, fetal health, socio-economic status, and the general condition of a country’s healthcare system.

### Prevalence of obesity and MetS due to epigenetic mechanisms

Another mechanism that might explain the “unexpected” prevalence of obesity and MetS is the epigenetic one. The environment is seen in epigenetics as a key player, and genes are the target of its actions. The focus is switched from gene to environment [20]. Some experts in the field of epigenetics consider that psychological distress (child abuse, for example) or deportation are events that might influence the way our genes are expressed. Others stand that nutrition could be another key factor able to modify and to pass to future generations a pattern of DNA methylation [17, 21-23].

There was a great debate in the medical literature regarding the role of poor maternal nutrition or psychological distress in offspring health. We are now in possession of a significant amount of data that demonstrates the detrimental effects of maternal distress on fetal and future adult development [24-26].

Studies have shown that exposing the developing fetus to maternal undernutrition or obesity increased the risk of obesity and diabetes during the adult life of the individual. Offsprings exposed to environmental stressors during early life have sustained alterations in cellular function and transcriptional regulation, suggesting developmental or epigenetic mechanisms that correlate with alterations in DNA methylation and/ or histone marks in rodent models [15, 27]. Unfortunately, the metabolic risk can be “transmitted” to subsequent generations, even in the absence of further environmental stressors. The idea of a relationship between perinatal nutrition and later long-term health was first hypothesized by Barker in 1997 [28] and later confirmed by numerous other studies [29, 30, 31].

Another important research field in epigenetics refers to psychological factors and their impact on the offspring. In rodents, it seems that early adversity has specific effects on the development of emotional learning systems [32, 33].

In humans, maternal depression has been associated with an increase in glucocorticoid receptor exon 1F region (GR-1F) promoter methylation in fetal blood, and this methylation pattern predicted hypothalamic–pituitary–adrenal (HPA) axis reactivity by the age of 3 months [34-38]. Maternal deprivation determined greater methylation of genes involved in the control of immune response and brain development and function in the case of institutionalized children [39, 40].

### Future directions. Proposals for the improvement of healthcare policies

Previous studies regarding the Effect of Traumatic Events on Populations (ETEP’s) involved hundreds or thousands of individuals. We assumed that almost the entire population of a country was exposed to highly traumatic events for an extended period.

Our statistical data confirm a brief, initial tendency for a rise of incidence in obesity and cardiovascular and metabolic diseases in the ‘70s. The instauration of the food rationing program during the ‘80s cut abruptly any increased tendency of metabolic diseases.

Generations were born in the ‘40s with a genetic program of food shortage, and the program proved to be correct, taking into account the latter evolution of ordinary Romanian citizens’ lifestyle during the Communist oppression. Children were also born in the ‘80s from malnourished mothers (especially in urban areas), and they were also welcomed by a harsh environment. However, this time, it was not for a long period of time.

The fall of the communist regime in 1989 turned upside down any “epigenetic programming”. Food became available, even in greater amounts than necessary. Romanian agriculture is highly performant today, one of the most competitive in Europe. Again, the epigenetic “program” turns to be wrong. We are now facing an obesity epidemic (the aggregate indicator for obesity level being three times higher compared to 1975), together with an explosive rise in metabolic and cardiovascular disease. There is a clear correlation between ETEPs from ’46-’47, ‘80s, and the burden of chronic disease of today.There are several hypotheses explaining the current situation. Maybe one of the best fittings is the “thrifty phenotype” hypothesis, which stated that “the poorly nourished mother essentially gives the fetus a forecast of the nutritional environment into which it will be born” [41-46].

The fetus must adapt “in utero” to the difficult conditions that will be encountered. Brain development is critical for such an adaptation; that is why the scarce nutrients are drained to the brain. “Unessential” organs such as the liver, muscles, or kidneys are sacrificed in favor of brain development. The adaptation process is epigenetic, involving changes in DNA methylation, histones, non-coding RNAs. Some of the most studied mechanisms imply insulin-like growth factor-II (overexpressed by hypomethylation in exposed embryos), with significant consequences in later insulin resistance, the emergence of obesity, or metabolic syndrome.

Another mechanism involves the disruption of the normal HPA axis. The low placental activity of 11β-hydroxysteroid dehydrogenase type 2 (11β HSD2) due to maternal malnourishment will lead to fetal overexposure of maternal corticoids. There will be important metabolic effects on the offspring and important psychiatric comorbidities due to impairment of the HPA axis [47, 48].

## Conclusions

The demonstration of an epigenetic mechanism driving the burden of diseases in Romania remains a goal for the future. There is abundant literature data regarding the role of physical training in reducing the incidence of various diseases, including dementia or metabolic syndrome. In rodents, ensuring an environmental enrichment combined with adequate physical training improves brain functioning and cognition throughout the lifespan of the animals [49, 50].

In this context, we have the scientific tools to consider the Romanian population at high-risk due to physical and psychological distress over long periods. The above model can be applied to any population that underwent harsh dictatorships and famine. It is now predictable that consumer behavior, considering what we called ETEPs, will lead to dramatic consequences in the prevalence of cardiovascular and nutritional diseases, especially metabolic syndrome or MAFLD.

Lessons should be learned from such experiences; consumer behavior must be balanced, educated, physical training must be encouraged through governmental programs in order to reduce the impact of chronic diseases on healthcare systems, as we know that health education can determine people to make informed decisions about their health, contributing to the evolution of the whole society [51]. No developing economy, no matter how fast it grows, can fully support the excessive burden of chronic diseases after long periods of deprivation. The good news regarding even “the thrifty phenotype hypothesis” is that after 2-3 generations without ETEPs, the population will adapt to the new, secure, abundance situation, and the burden of chronic diseases will decrease.

Unfortunately, even though MAFLD has an increasing prevalence worldwide, becoming the main cause of liver transplantation in the United States, with estimated lifetime costs for nonalcoholic steatohepatitis patients of $222.6 billion in 2017, with an estimated prevalence in Europe of 23%, there are no national and regional government strategies addressing MAFLD except for Germany, Italy, Spain, and United Kingdom [52].

There might be some unexpected consequences for populations with a high prevalence of MAFLD. For example, in the case of a pandemic such as COVID-19, it seems that obesity-related fatty liver disease increases the risk of severe infection by six-fold. We should not forget that adipose tissue is transformed into a reservoir of COVID-19; obese patients already have a pro-inflammatory condition, which could be exacerbated by the virus, leading to a dangerous multisystem inflammatory syndrome [53-55].

From this point of view, the Romanian population is also at high risk, and the recent evolution of the COVID-19 pandemic in Romania seems to confirm such a hypothesis. The present research can happily be complemented by at least two future directions of research regarding the link between MAFLD and the influence of epigenetic mechanisms on the one hand and MAFLD and the risk associated with COVID 19 disease on the other hand.

## Conflict of Interest

The authors declare that there is no conflict of interest.
